# Case of Congestion and Softening of the Brain

**Published:** 1861-07

**Authors:** R. R. McMeens

**Affiliations:** Sandusky, Ohio


*Case of Congestion and Softening of the Brain.* By R. R. McMeens, M. D., Sandusky, Ohio.

The prodroma, progress, and peculiar symptoms of the following case were so novel in their nature, to myself and others, as to induce me to submit an abstract of the same to your perusal, and if considered of sufficient interest to warrant publication, you can so dispose of it.

D. D., an employee of the S., D. and C. R. R., had occupied the post of dock and road master at this place for the last fifteen years. He possessed great physical energy and durance, and was indefatigably diligent in discharging the duties of his station. He was of a nervo-bilious temperament, of medium stature, modest demeanor, and acknowledged probity of character. During the last ten years, the period of my acquaintance with, and professional cognizance of him, he had suffered from but two attacks of illness: some six years, from an acute pneumonia, induced by exposure at night to inclement weather, from which he had a rapid and perfect recovery; and about a year subsequently, from a slight hæmoptysis, excited by over-exertion and exercise, while suffering from a severe pulmonary catarrh. With these exceptions, his health had been uniformly good, requiring no medical aid whatever; nor interrupting the regular performance of his duties, until late in the summer of last year, when he began to complain of feeling unusually fatigued after the labors of the day, and was observed by his intimate associates to incline his head unnaturally to one side, and to perceptibly droop in one shoulder. He made an allusion, however, to this fact himself, and was apparently unconscious of it. In September last he was compelled to undergo unusual labor, and exercise great vigilance in providing and superintending the numerous trains required to accommodate and transport the people attending the "mass meetings" of the late political campaign. On the 23d of that month he stood most of the night on the platform of the rear car, with a signal light, to prevent any casualty occurring between the many "wild trains" then in transition. The next day, although weary from want of sleep, and suffering from soreness, and stiffness in the shoulders, arms, and back of the neck, which he attributed to rheumatism, he accompanied a monster train to the grand gathering of the "Douglas clan" at Tiffin. Before reaching that place he was seized with chills, flashes of fever, increased pain and lameness, thirst, dyspnœa, and extreme difficulty and distress in swallowing. At Tiffin he proceeded at once to a hotel and retired to bed, where he remained until the return of the train, with which he arrived at home early in the evening. I was immediately summoned to his assistance, and found him suffering apparently from most of the prominent symptoms of a congestive chill, common to our pernicious fevers, aud diagnosed accordingly. The pulse was accelerated, contracted, and quick, oppressed respiration, cold and clammy surface, anxious and haggard countenance, slightly coated tongue, and a general tremor of the whole body. There was intense thirst, but an almost total inability to swallow any fluid, it being spasmodically ejected from the nostrils, or attended with symptoms of impending suffocation. He was also tormented with frequent shocks or distressing sensations in the arms and hands, requesting them to be vigorously pulled, rubbed, and pinched.

A mustard poultice was premised, and a large cataplasm of the same directed to be applied over the whole surface of the chest and the following prescription ordered:

℞ Quiniæ sulph., ℈ j.
Pulv. Doveri, grs. xxv.
M., ft. chart. No. iv. S. one every four hours,

To be alternated with teaspoonful doses of spirits nit. dulc. in water. After a time he felt somewhat relieved, but passed an unquiet and unsatisfactory night.

Sept. 25.—Morning visit: Respiration much improved, pulse less frequent and more natural, deglutition still difficult, but practicable, tongue more coated, surface warm and normal. Patient complains chiefly of severe and circumscribed pain in the left side of the thorax. Percusion elicits slight dullness over the region of pain. Ordered he free application of cups over the affected part, and made the following prescription:

℞ Quiniæ sulph., ℈ j.
Hydrarg. massæ, grs. xij.
Pulv, ipecac., grs. iv.
Extract hyosciamus, grs. vj.
M., ft. pill. No. xij. S. one every two hours.

Spirits nit. dulc. to be continued as before.

Evening visit: Respiration and deglutition decidedly improved. Pain in chest quite trivial. Patient expresses a general sense of relief. Has had no evacuation of the bowels; some uneasiness in consequence. Ordered a bolus of the following:

℞ Hydrarg. submur., grs. viij.
Extract hyosciamus, grs. ij. M.

To be followed in four hours with an infusion of—

℞ Senna, ℥ ss.

Magnes. sulph., ʒ ss.

Mannæ opt., ℥ j.

Fœnicula contus., ʒ ij.

M. To be given in free doses every two hours until it operates.

Sept. 26.—Morning visit: Cathartic had acted freely, early in the night; has slept but little; passed a restless but painless night. Complains of slight pain and uneasiness in the occiput and nape of the neck. Pulse quite natural. Some difficulty of swallowing occasionally experienced; at other times perfectly free. Some degree of numbness and uneasiness of the upper extremities returning. Observe for the first time an unnatural lustre of the eyes, and some dilatation of pupils. Prescribed the following as a nervous stimulant:

℞ Assafœtida, ʒ j.

Aqua menthæ, ℥ iij. Ft. solutio, et adde

Ammoniated tinct. valerianæ, ʒ j.

Tinct. castorei, ʒ ij.

Sulphuric ether, ʒ j.

Tinct. opii camph., ℥ ss.

M. Teaspoonful every two or three hours.

Evening visit: Informs me confidentially of a new feature in his case: has had, all the afternoon, frequent violent and painful erections of the penis, attended with emission of semen, which he feels is rapidly exhausting him; has been vainly endeavoring to restrain them by the application of cold water. Both himself and attendant concur in assuring me that he has had more than a dozen emissions already. While conversing with him another erection suddenly and unexpectedly occurred, which he ineffectually attempted to arrest by force of will. To satisfy myself of the real character of this singular phenomenon I made an occular inspection of the organ. The penis was in a state of full and complete priapism, the glans purple from extreme distention, the testicles firmly contracted, and the emission forcibly ejected, but small in quantity. After its consummation, an excessive degree of languor ensued, with general muscular tremor and frequent and feeble pulse, and evident marks of exhaustion.

A blister was applied to the nape of the neck, and a liniment, composed as follows, addressed to the spine, by friction:

℞ Volatile liniment, ℥ iv.

Spirits terebinth., ℥ ss.

Tinct. opii, ℥ ss. M.

And the following sedative administered internally, to quiet general nervous perturbation:

℞ Chloroform, gtts. x.

Tinct. opii, ʒ ij.

Mucilago acaciæ, ℥ ss.

Aq. laura cerasi, ℥ ss.

Aq. distillata, ℥ j.

M. Teaspoonful every two or three hours.

The application of cold water to the parts to be continued.

Sept. 27.—Was called at 2 o'clock, A. M. Patient worse. Erections still frequent, emissions only occasional; extreme wakefulness; increasing cerebral excitement, but intellectual functions still unimpaired; the erections are becoming intolerably painful, causing him to cry out with anguish. Continue same treatment until morning, with additional doses of the muriate of morphia combined with the extract of dulcamara.

Morning visit: Patient no better. Symptoms much the same. The erections only partial, and of shorter duration; they assume more the character of neuralgic shocks, and are exquisitely painful. He inquired as to the probable prospects of his recovery. I confessed my apprehension of the result, and solicited counsel. He immediately sent for an attorney, and made his will with a composure and equanimity of mind rarely witnessed under similar circumstances. I then secured the counsel and coöperation of Dr. H. I. Donahoo, and we placed him upon the following treatment:

℞ Extract nux vomica, grs. vj.

Fluid extract conii, ℥ ij.

M. Teaspoonful every two or three hours.

Toward evening he began to manifest some aberration of mind, become abstracted, and, if unmolested, mutter to imaginary persons; all of which, however, would disappear upon his attention being diverted by interrogation or otherwise. During the night his delirium and hallucinations rapidly increased, the current of which was decidedly libidinous in character. He was constantly engaged in contracting secret assignations with numerous and voluptuous syrens. His language, at times, was lewd to the extreme of obscenity.

Sept. 28.—Patient more excited and incoherent, the character of his hallucinations less lecherous, but more hideous and harrassing. He imagines himself incarcerated and condemned for seducing the sister of one of his friends, and pleads vehemently and most pathetically in asseveration of his innocence. About noon a complete change came over the *phantasia* of his dreams; the wanton fires that had raged so fiercely in the chambers of reason, were extinguished, and all traces of their imagery had vanished as the shadow of a cloud. The organs of mirth and benevolence seemed to have gained possession, and reign supreme. He winked at all within the reign of his vision, with a most benignant and winning smile, insisted upon treating the whole crowd, and was exceedingly jocose, and lavish in offers of money and munificent gifts.

During all this time an occasional shock or nervous paroxysm would pass over him, momentarily check his hilarity, produce a painful contortion of countenance, and cause him to clasp his hands firmly upon the pubis. These shocks closely resembled in their conduct the action of a person subjected to the charge of a galvanic battery. He continued in this state until about five o'clock in the afternoon, when, in the midst of one of his happiest moods and broadest smiles, another convulsive shudder occurred, respiration ceased, and, with one spasmodic convulsion of the face, he expired.

The next morning a hurried post-mortem was had, in the presence of Drs. Donahoo, Austin Morton, and Stanley. Upon removing the calvarium the meninges exhibited an undue degree of vascularity, and the sinuses engorged with an exceedingly dark-colored blood. On exposing the brain proper, the anterior lobes of the cerebrum presented a perfectly normal appearance, while, upon the extreme portion of the posterior lobes a distinct line of demarcation was conspicuously displayed between the pearly and pink-colored tint of the healthy structure, and the dingy, degenerated aspect of the other. It presented precisely the appearances observed in cases of chronic softening. The whole exterior of the cerebellum, with the pons varolii and medulla oblongata, were found in a state of intense vascular congestion. No further examination was made, as time and place prevented the proper opportunities for minute exploration.

The autopsical revelations detailed above, forces me to the conclusion that chronic softening of the brain had been silently encroaching for some time previous to the last attack, and that the local character and predilection of the cerebellum, as the seat of congestion, was the consequence of preëxisting disease and debility in that region.

To explain the peculiar phenomena displayed in the case, would require all the learning and acumen of the most profound physiologist. The singular fact that the nervous centre presiding over the sexual organs, should be specially elected and excited to such an inordinate degree, and to the exclusion of all others, as directly under the control of the cerebro-spinal system, as well as the eccentric paralyzation of the nerves concerned in the process of deglutition, with the paroxysmal affection of the upper extremities, and subsequently invading the intellectual faculties, developing a series of mental manifestations almost purely phrenological in proportion and propensity, demands the attainments of a Brown-Sequard or Fraser Campbell to fully comprehend and rationally demonstrate.—*Lancet & Observer.*

